# Exploring the feasibility of FOCUS DWI with deep learning reconstruction for breast cancer diagnosis: A comparative study with conventional DWI

**DOI:** 10.1371/journal.pone.0313011

**Published:** 2024-10-31

**Authors:** Yue Ming, Fan Yang, Yitian Xiao, Shuting Yue, Pengfei Peng, Xun Yue, Qian Pu, Huiyi Yang, Huilou Liang, Bo Zhang, Juan Huang, Jiayu Sun

**Affiliations:** 1 Department of Radiology, West China Hospital, Sichuan University, Chengdu, Sichuan, China; 2 Department of Radiology, Affiliated Hospital of North Sichuan Medical College, Nanchong, Sichuan, China; 3 GE HealthCare MR Research, Beijing, China; Ascension Sacred Heart Hospital Pensacola, UNITED STATES OF AMERICA

## Abstract

**Purpose:**

This study compared field-of-view (FOV) optimized and constrained undistorted single-shot diffusion-weighted imaging (FOCUS DWI) with deep-learning-based reconstruction (DLR) to conventional DWI for breast imaging.

**Methods:**

This study prospectively enrolled 49 female patients suspected of breast cancer from July to December 2023. The patients underwent conventional and FOCUS breast DWI and data were reconstructed with and without DLR. Two radiologists independently evaluated three images per patient using a 5-point Likert scale. Objective evaluations, including signal-to-noise ratio (SNR), contrast-to-noise ratio (CNR), and apparent diffusion coefficient (ADC), were conducted using manual region of interest-based analysis. The subjective and objective evaluations were compared using the Friedman test.

**Results:**

The scores for the overall image quality, anatomical details, lesion conspicuity, artifacts, and distortion in FOCUS-DLR DWI were higher than in conventional DWI (all *P* < 0.001). The SNR of FOCUS-DLR DWI was higher than that of conventional and FOCUS DWI (both *P* < 0.001), while FOCUS and conventional DWI were similar (*P* = 0.096). Conventional, FOCUS, and FOCUS-DLR DWI had similar CNR and ADC values.

**Conclusion:**

Our findings indicate that images produced by FOCUS-DLR DWI were superior to conventional DWI, supporting the applicability of this technique in clinical practice. DLR provides a new approach to optimize breast DWI.

## Introduction

As of 2020, breast cancer is the most diagnosed cancer in women, accounting for ~2.3 million new cases [1]. According to data up to 2023, it constituted 31% of female cancers and was a leading cause of cancer-related deaths in women aged 20–49 [2]. Early diagnosis is crucial for better prognosis and survival rates [3].

MRI is commonly used for breast cancer diagnosis due to its high sensitivity in detecting malignant breast tumors [4]. Diffusion-weighted imaging (DWI) is an MRI technique that does not require a contrast agent. It can serve as a complement to dynamic contrast-enhanced MRI or, in specific cases, replace it. DWI plays a significant role in diagnosing breast cancer, predicting lymph node metastasis, assessing prognosis, and predicting outcomes of neoadjuvant chemotherapy [5–7].

Conventional DWI is based on a single-shot echo-planar imaging sequence, offering advantages such as motion robustness and high signal-to-noise ratio (SNR) efficiency [8, 9]. However, the narrow effective bandwidth in the phase-encoding direction of the single-shot planar echo-planar imaging sequence makes it susceptible to off-resonance effects and sensitive to magnetic field inhomogeneity, leading to image artifacts and distortions [10, 11]. Furthermore, this sequence exhibits noticeable T2* decay during the echo-planar imaging readout, resulting in low resolution, geometric distortions, and image blurring [12].

To address these challenges, the field-of-view (FOV)-optimized and constrained undistorted single-shot (FOCUS) technology was introduced into DWI as a novel sequence [13]. FOCUS DWI utilizes a 2D spatially-selective echo-planar radiofrequency excitation pulse to reduce the FOV in the phase-encoding direction, thereby decreasing the number of phase-encoding lines and readout time, improving the DWI image quality with reduced magnetic susceptibility and fewer motion artifacts [14, 15]. Previous studies conducted in small anatomical areas, such as the prostate, pancreas, and rectum, have demonstrated that FOCUS DWI was superior to conventional DWI in lesion conspicuity, image artifacts, blurring, distortion, and overall image quality [14, 16–21]. Previous studies have also shown that small-field DWI can achieve high image resolution and reduced image distortion in breast imaging, allowing for better lesion visualization [10, 22–25]. FOCUS DWI can effectively display all glandular tissues and axillary lymph nodes for small breast sizes. Although artifacts and distortions are reduced, the smaller excitation FOV in FOCUS DWI results in a consistently lower image signal-to-noise ratio (SNR) [18, 21, 26].

Deep learning algorithms offer a promising solution to addressing this challenge. Recently, an innovative deep learning-based reconstruction (DLR) method employing a deep convolutional neural network (CNN) has been developed to reduce noise and improve image quality [27]. This technique directly reconstructs k-space data to generate high-quality images with reduced noise, minimized truncation artifacts, and improved sharpness. This DLR method has been applied across various MRI applications, including joints, muscles, prostate, and bladder, consistently demonstrating improvements in image quality and diagnostic accuracy [28, 29]. Previous studies have shown that the DLR can improve the lesion edge delineation and internal features in DWI images, and can also supplement clinical staging and improve the sensitivity and effectiveness of early diagnosis [30, 31].

The high level of noise and fairly low SNR in conventional DWI images may complicate the differentiation of physiological breast tissue from tumor lesions in breast DWI [32]. Although FOCUS DWI can reduce artifacts and distortion compared to conventional DWI, its images still have a lower SNR. We hypothesized that DLR could improve the SNR and clarity of FOCUS DWI images, thereby facilitating the detection of breast lesions. Therefore, this study aimed to evaluate the feasibility of employing FOCUS DWI with DLR (FOCUS-DLR) in the assessment of breast cancer. We assessed the impact of DLR on image quality as well as on parameters such as the apparent diffusion coefficient (ADC), SNR, and contrast-to-noise ratio (CNR), both qualitatively and quantitatively, by comparing FOCUS, FOCUS-DLR, and conventional DWI.

## Materials and methods

### Study subjects

**Fig 1:**
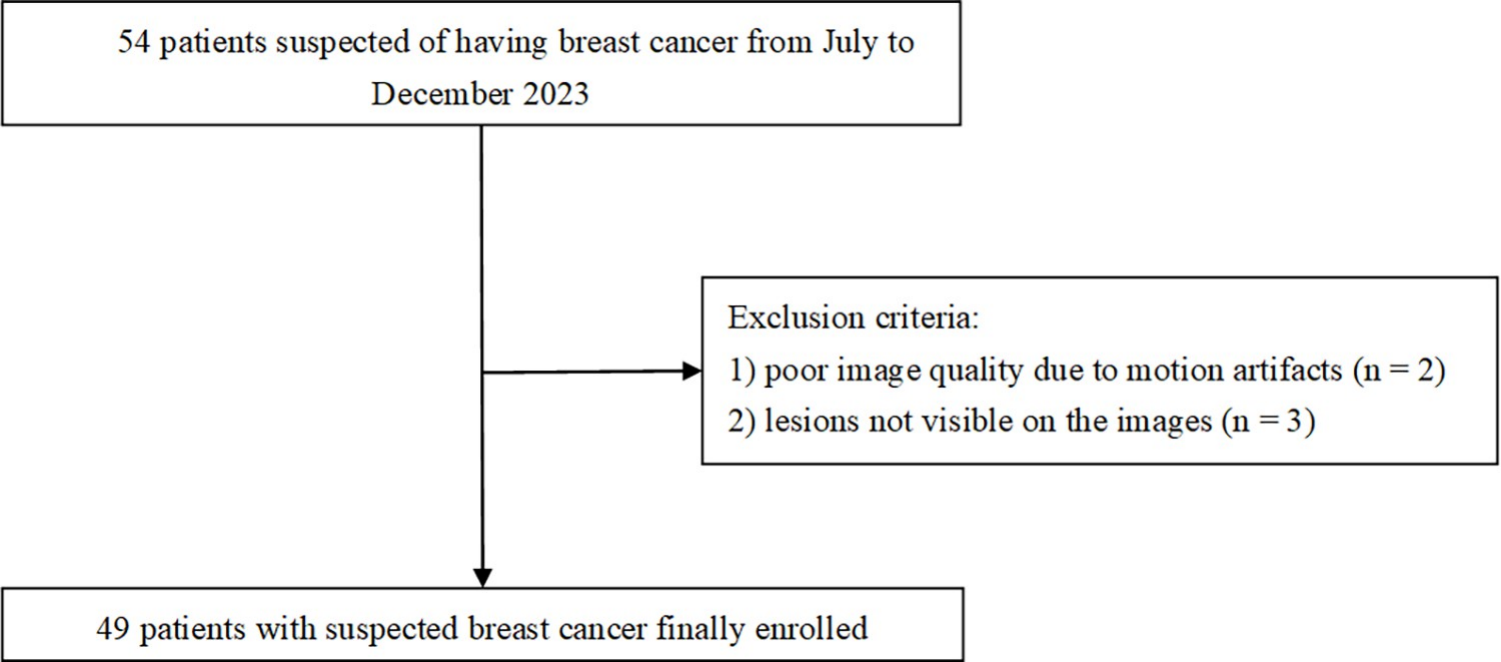
Flowchart of patient selection.

Ethical approval for the study was obtained from the Biomedical Ethics Review Committee of West China Hospital, Sichuan University (No. 187). Written informed consent was obtained before the inclusion of patients. This study enrolled female patients with suspected breast cancer at West China Hospital, Sichuan University from July to December 2023. Inclusion criteria were as follows: (1) patients with complete clinical data; (2) patients who underwent MR examinations, including conventional DWI, FOCUS DWI, and FOCUS-DLR DWI. Exclusion criteria included: (1) poor image quality due to motion artifacts; (2) lesions not visible on the images; (3) the breast volume is too large, exceeding the FOV of focus DWI. A flowchart showing the recruitment process is shown in Fig 1.

### MRI protocol

All breast MRI acquisitions were performed on a 3.0T MR scanner (SIGNA Premier, GE Healthcare, Milwaukee, WI) with a dedicated 8-channel bilateral breast coil. The patients were in a prone position with feet first for bilateral breast MRI examination. The clinical breast MRI protocol was extended to include conventional single-shot and FOCUS DWI, with b-value of 0 and 1,000 s/mm^2^ [33] and the same scan coverage. The imaging parameters for both sequences were carefully selected based on prior studies and our own institutional experience to balance image quality, acquisition time, and patient comfort. The detailed imaging parameters for the conventional DWI were: FOV = 340 mm×340 mm; auto repetition time (TR) = 4,525 ms; minimum echo time (TE) = 53.4 ms; number of slices = 32; matrix size = 128×168; number of excitations = 4 (b1000); voxel size = 2.7×2.0×4.0 mm; bandwidth = 3,906.25 Hz/Px; parallel imaging (ASSET) factor = 2; acquisition time = 127 s. The FOCUS DWI used similar imaging parameters as follows: FOV = 340 mm×170 mm; auto TR = 10,079 ms; minimum TE = 51.0 ms; number of slices = 32; matrix size = 128×84; number of excitations = 4 (b1000); voxel size = 2.7×2.0×4.0 mm; bandwidth = 3,906.25 Hz/Px; no parallel imaging; acquisition time = 143 s. The longer auto TR for FOCUS DWI is attributed to the use of a 2D RF excitation pulse. Additionally, parallel imaging was not employed for FOCUS DWI due to the reduced field of view (FOV) in the phase encoding direction [20].

### Data processing

The DLR is commercially known as AIR^TM^ Recon DL (GE Healthcare). It is an algorithm in the MR image reconstruction pipeline that utilizes a cascade of more than 100,000 distinct pattern recognitions for noise and low resolution to reconstruct the optimal object image [27]. The highly optimized Convolutional Neural Network algorithm was integrated into the MR image reconstruction pipeline to process original k-space data directly, producing DLR images with reduced noise and truncation artifacts. This integration aims to shorten scanning time and enhance overall image quality [34]. The network features an adjustable SNR enhancement level tailored to the user’s preferences and an innovative technology for suppressing ringing artifacts. This technology identifies typical artifacts such as Gibbs ringing and truncation and transforms them into enhanced image details. The outcome is an image characterized by high SNR and spatial resolution [35].

ADC map reconstruction was performed on the vendor-supplied workstation (Advantage Workstation Ver 47, GE Healthcare) using a mono exponential fitting based on DWI images with b-values of 0, and 1000 s/mm^2^.

### Image analysis

**Fig 2:**
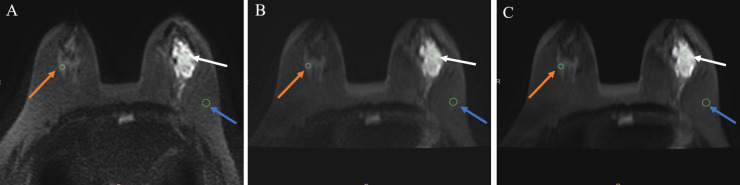
A 51-year-old female with breast cancer in the left breast. A, conventional DWI; B, FOCUS DWI; C, FOCUS-DLR DWI. This image shows the delineation of the ROIs, where the white arrow indicates the lesion ROI, the blue arrow indicates the background ROI, and the orange arrow indicates the glandular tissue ROI. DLR, deep-learning-based reconstruction; DWI, diffusion-weighted imaging; FOCUS, field-of-view optimized and constrained undistorted single-shot; ROI, region-of-interest.

Two radiologists with 3, and 10 years of working experience in breast MRI, unaware of the DWI sequence details, independently evaluated the image quality. Images with a b-value of 1,000 s/mm^2^ from each DWI sequence were used for qualitative image analysis. These images were randomly assigned to the radiologists. A 5-point Likert scale (1 = Poor, 5 = Excellent) was employed to assess the overall image quality, display of anatomical details, lesion conspicuity, artifacts, and geometric distortions [16, 18, 21] (S1 Table). For quantitative evaluation, the radiologists outlined the lesions and calculated the SNR and CNR between the lesion and surrounding tissue. Radiologists also measured the lesion ADC values. Regions of interest (ROIs) were delineated on the lesion’s maximum cross-sectional plane. Surrounding tissue ROIs were placed on healthy glandular tissue, while background ROIs were drawn in areas of uniform fat at the same plane [15] (Fig 2).

The SNR and CNR were calculated based on the following formulas [36, 37]:

$$SNR=\frac{{SI}_{lesion}}{{SD}_{background}}$$

$$CNR=\frac{\left|{SI}_{lesion}-{SI}_{tissue}\right|}{\sqrt{{{SD}_{lesion}}^{2}+{{SD}_{tissue}}^{2}}}$$

where: SI_lesion_ and SI_tissue_ are the average signal intensity (SI) of the lesion and surrounding ROIs, respectively; SD_background_, SD_lesion_, and SD_tissue_ are the standard deviation (SD) of the background, lesion, and surrounding ROI SI values, respectively.

The lesion ADC value was obtained by drawing an ROI on the area corresponding to the lesion on the ADC map. The ROI should be entirely within the lesion, avoiding artifacts and necrotic or hemorrhagic areas. The lowest ADC value in the lesion can most accurately differentiate between malignant and benign breast lesions [38]. Therefore, in this study, we placed the ROI on the darkest part of the lesion on the ADC map when measuring the ADC value [39].

### Statistical analysis

Data were analyzed using IBM SPSS Statistics for Windows, Version 20.0 (IBM Corp., Armonk, NY). The normality of continuous data was assessed using the Kolmogorov-Smirnov *D* test, and histograms were employed for visual inspection. Normally distributed data are presented as means ± SD, while skewed data are presented as medians (interquartile ranges). Categorical data are expressed as percentages and frequencies. Differences in evaluations among multiple sequences were compared using one-way analysis of variance (ANOVA) or Friedman test, with Dunn-Bonferroni post hoc tests applied to adjust for multiple pairwise comparisons. Inter-observer consistency for qualitative metrics was evaluated using the Cohen’s Kappa (κ) test, with Kappa of 0.41–0.60 considered moderate, 0.61–0.80 substantial, and > 0.81 considered almost perfect agreement. Intra-observer and inter-observer consistency for quantitative metrics was assessed using the intraclass correlation coefficient (ICC). ICC values under 0.5 were considered poor, between 0.5 and 0.75 moderate, between 0.75 and 0.9 good, and over 0.9 as excellent reliability [40]. Statistical significance was set at *P* < 0.05.

## Results

### Clinical characteristics

This study included 49 patients suspected of having breast cancer from July to December 2023, with an average age of 48.6 ± 10.2 (range 27–70) years. Among them, 41 (84%) had invasive carcinoma, six (12%) had ductal carcinoma *in situ*, one (2%) had a breast cyst, and one (2%) had a malignant phyllodes tumor.

### Qualitative analysis

**Fig 3:**
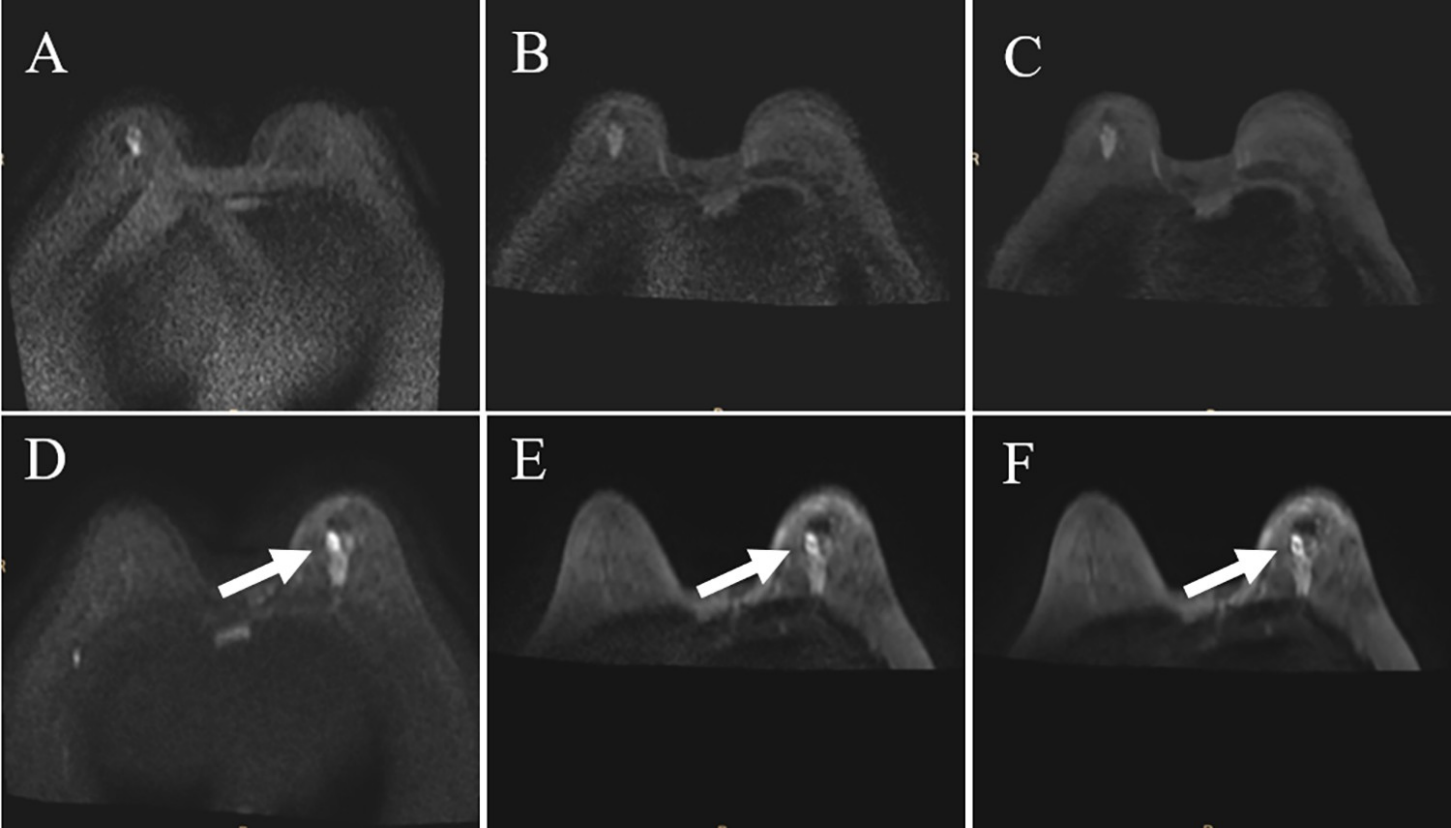
Images used for subjective quality assessment. Conventional (A), FOCUS (B), and FOCUS-DLR (C) DWI of a 64-year-old patient with suspected invasive breast cancer in the right breast. The overall image quality of FOCUS DWI was superior to that of conventional DWI and inferior to that of FOCUS-DLR DWI. Conventional (D), FOCUS (E), and FOCUS-DLR (F) DWI of a 49-year-old patient with suspected invasive cancer in the left breast. FOCUS (E) and FOCUS-DLR (F) DWI are superior to conventional DWI (D) in lesion conspicuity and anatomical detail, where the white arrow indicates the lesion. DLR, deep-learning-based reconstruction; DWI, diffusion-weighted imaging; FOCUS, field-of-view optimized and constrained undistorted single-shot.

**Fig 4:**
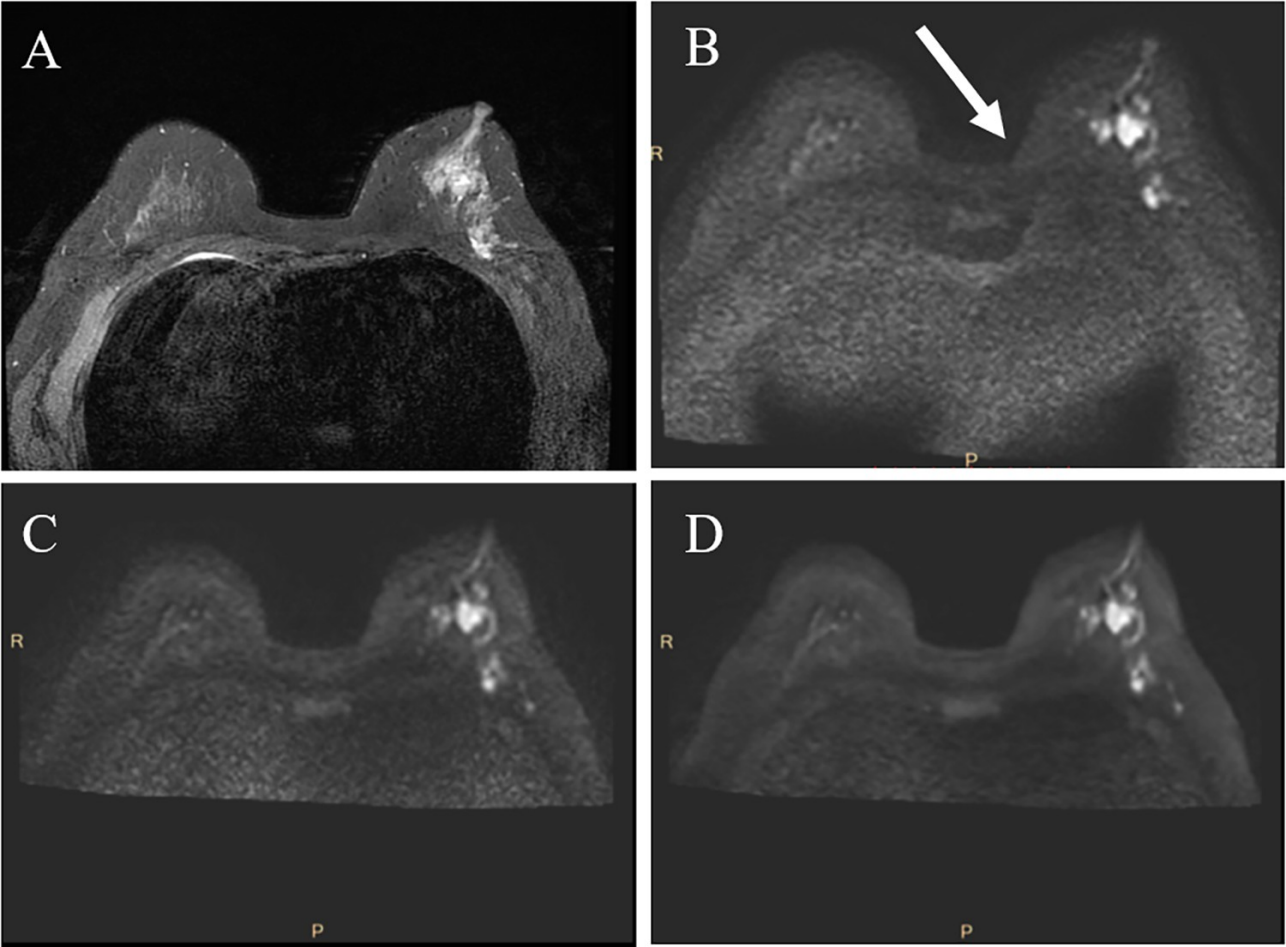
MR images of a 57-year-old patient suspected of invasive cancer in the left breast. A, T2-weighted imaging; B, conventional DWI; C, FOCUS DWI; D, FOCUS-DLR DWI. Conventional DWI exhibits noticeable ghosting, where the white arrow indicates the ghosting. However, FOCUS and FOCUS-DLR DWI show reduced ghosting. DLR, deep-learning-based reconstruction; DWI, diffusion-weighted imaging; FOCUS, field-of-view optimized and constrained undistorted single-shot.

The qualitative analysis found significant differences among the conventional, FOCUS, and FOCUS-DLR DWI in overall image quality, anatomical details, lesion conspicuity, artifacts, and distortion (Table 1). Examples are shown in Figs 3 and 4. Specifically, FOCUS and FOCUS-DLR DWI had greater anatomical detail, lesion conspicuity, artifact, and distortion scores than conventional DWI (all *P* < 0.001). Additionally, FOCUS-DLR DWI was pronouncedly superior to FOCUS DWI in overall image quality, anatomical detail representation, and lesion conspicuity (*P* < 0.001, *P* < 0.001, and *P* = 0.004, respectively).

**Table 1:** Subjective image quality assessment.

Qualitative parameter	Conventional DWI	FOCUS DWI	FOCUS-DLR DWI	*P*-value
Overall image quality	3.06±0.38	4.02±0.32^†^^,^***	4.82±0.39^†^^,^ ***^;^ ^‡^^,^***	< 0.001
Anatomical details	3.12±0.33	4.10±0.31^†^^,^***	4.90±0.31^†^^,^ ***^;^ ^‡^^,^***	< 0.001
Lesion conspicuity	3.14±0.35	4.02±0.14^†^^,^***	4.65±0.48^†^^,^ ***^;^ ^‡^^,^**	< 0.001
Artifacts	2.90±0.55	3.80±0.46^†^^,^***	4.08±0.40^†^^,^ ***	< 0.001
Geometric distortion	3.10±0.42	3.96±0.29^†^^,^***	4.16±0.43^†^^,^ ***	< 0.001

Data are presented as means ± SD

**P < 0.01

***P < 0.001

†vs. conventional DWI

‡vs. FOCUS DWI (Bonferroni post-hoc analysis). DWI, diffusion-weighted imaging; FOCUS, field-of-view optimized and constrained undistorted single-shot; DLR, deep learning-based reconstruction.

### Quantitative analysis

The SNR of FOCUS-DLR DWI was higher than that of conventional and FOCUS DWI (both *P* < 0.001), while the latter two were similar (*P* = 0.207; Table 2). The CNR values were similar in all three methods.

**Table 2:** Objective quantitative assessment of conventional, FOCUS, and FOCUS-DLR DWI.

Quantitative parameter	Conventional DWI	FOCUS DWI	FOCUS-DLR DWI	*P*-value
SNR	30.28 (19.76, 38.08)	37.90 (17.17, 47.51)	62.61 (29.20, 80.38)^†^^,^ ***^;^ ^‡^^,^***	< 0.001
CNR	3.48 (1.70, 4.84)	3.45 (1.89, 4.60)	3.44 (1.82, 4.63)	0.936
SI_lesion_	122.42 (71.57, 155.57)	107.36 (77.20, 132.81)^†^^,^*	107.18 (77.56, 134.15) ^‡^^,^*	< 0.01
SD_background_	4.17 (3.05, 5.18)	3.48 (2.46, 4.06)	2.29 (1.40, 2.44)^†^^,^ ***^;^ ^‡^^,^***	< 0.001
ADC	1.51(1.09, 1.97)	1.62 (1.01, 2.29)	1.61 (1.01, 2.28)	0.096

Data are given as medians (interquartile ranges).

*P < 0.05

***P < 0.001

†vs. conventional DWI

‡vs. FOCUS DWI (Bonferroni Post-hoc analysis). ADC, apparent diffusion coefficient; CNR, contrast-to-noise ratio; DWI, diffusion-weighted imaging; DLR, deep-learning-based reconstruction; FOCUS, field-of-view optimized and constrained undistorted single-shot; SD_background_, standard deviation of the background region-of-interest signal intensity; SI_lesion_, signal intensity in the lesion; SNR, signal-to-noise ratio.

The SI_lesion_ of FOCUS DWI was lower than that of conventional DWI (*P* = 0.035) but higher than that of FOCUS-DLR DWI (*P* = 0.006). The SD_background_ of FOCUS-DLR DWI was lower than that of conventional and FOCUS DWI (both *P* < 0.001). No difference was found among the methods in their mean ADC values (Table 2).

### Image quality interobserver reliability

**Fig 5:**
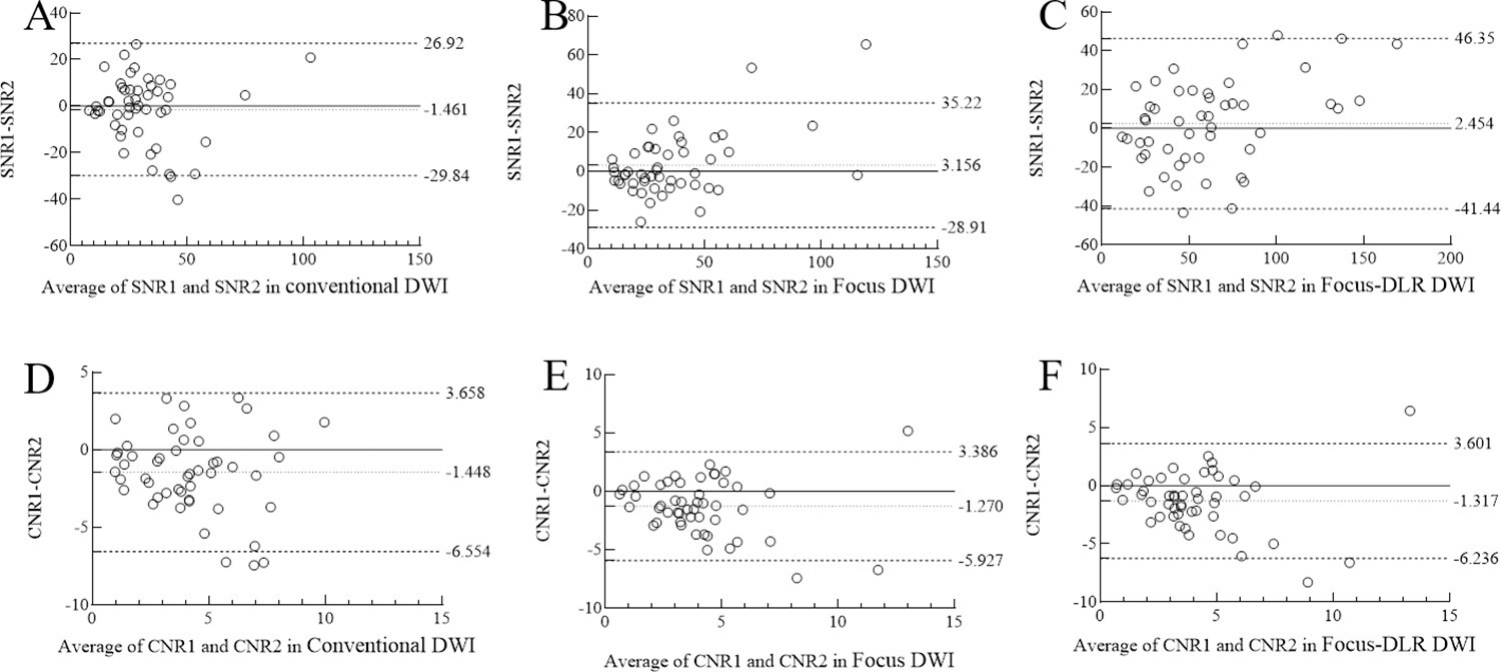
Interobserver agreement on SNR for conventional (A), FOCUS (B), and FOCUS-DLR (C) DWI. Interobserver agreement on CNR for conventional (D), FOCUS (E), and FOCUS-DLR (F) DWI. The solid line represents the level where the difference between the two readers was zero, the central dashed line represents the mean difference, and the upper and lower dashed lines represent the 95% limits of agreement. CNR, contrast-to-noise ratio; DWI, diffusion-weighted imaging; DLR, deep-learning-based reconstruction; FOCUS, field-of-view optimized and constrained undistorted single-shot; SNR, signal-to-noise ratio.

For conventional DWI, we found a relatively Poor agreement in all subjective image quality scores (overall image quality, anatomical details, lesion conspicuity, artifacts, and distortion), with interobserver κ values of 0.232–0.662 (S2 Table). A fairly high degree of agreement was observed in subjective image quality scores for FOCUS and FOCUS-DLR DWI, with interobserver κ values of 0.635–0.871 (S2 Table). The inter- and intra-observer agreement on the SNR and CNR of the conventional, FOCUS, and FOCUS-DLR DWI was moderate to good (ICC > 0.400; Table 3). The Bland-Altman Plots for the inter-observer agreement on the objective image quality values (SNR, CNR) are shown in Fig 5. For conventional DWI, 89.8% (44/49) and 93.9% (46/49) of the values, respectively, fell within the 95% range. The respective values for FOCUS DWI were 95.9% (47/49) and 93.9% (46/49), and for FOCUS-DLR DWI, they were 91.8% (45/49) and 93.9% (46/49). The ADC reliability for conventional, FOCUS, and FOCUS DLR DWI was moderate to excellent (ICC > 0.730; Table 3).

**Table 3:** ICC values for inter- and intra-observer agreement on the SNR and CNR of conventional, FOCUS, and FOCUS-DLR DWI.

	Conventional DWI	FOCUS DWI	FOCUS-DLR DWI
Inter-observer agreement			
SNR	0.682 (*P* < 0.001)	0.790 (*P* < 0.001)	0.833 (*P* < 0.001)
CNR	0.404 (*P* < 0.001)	0.540 (*P* < 0.001)	0.509 (*P* < 0.001)
ADC	0.818 (*P* < 0.001)	0.731 (*P* < 0.001)	0.742 (*P* < 0.001)
Intra-observer agreement			
SNR	0.548 (*P* = 0.03)	0.798 (*P* = 0.002)	0.713 (*P* = 0.009)
CNR	0.626 (*P* = 0.016)	0.828 (*P* < 0.001)	0.695 (*P* = 0.004)
ADC	0.906 (*P* < 0.001)	0.919 (*P* < 0.001)	0.921 (*P* < 0.001)

ADC, apparent diffusion coefficient; CNR, contrast-to-noise ratio; DWI, diffusion-weighted imaging; DLR, deep-learning-based reconstruction; FOCUS, field-of-view optimized and constrained undistorted single-shot; SNR, signal-to-noise ratio.

## Discussion

This study presents evidence suggesting that FOCUS-DLR DWI offers image quality superior to both FOCUS and conventional DWI. Our comparative image quality analysis also revealed that FOCUS DWI outperformed conventional DWI in the subjective evaluations. Furthermore, our study demonstrated a noteworthy enhancement in image quality, particularly in the overall image quality, anatomical details, and lesion conspicuity, when using the DLR technique within the FOCUS DWI framework. Notably, the SNR in FOCUS-DLR DWI was significantly higher than in FOCUS and conventional DWI. We found no difference in ADC values across the three modalities, suggesting consistency in their quantitative metrics.

The proximity of the breast to the thoracic cavity makes breast DWI susceptible to motion artifacts caused by physiological movements such as breathing and heartbeat. Additionally, the breasts deviate from the magnetic field center due to their shallow anatomical position, and a significant amount of air is present in the surrounding area [24]. Conventional DWI sequences based on echo planar imaging are highly sensitive to magnetic field inhomogeneity and physiological motion, especially in breast imaging, leading to distortion and artifacts. The FOCUS DWI technology reduces the FOV in the phase-encoding direction and the number of k-space lines to enhance image resolution and reduce distortion and artifacts while maintaining a clinically acceptable scan time [41, 42]. The results of this study indicated that both FOCUS and FOCUS-DLR DWI produced higher-quality images than conventional DWI. They outperformed conventional DWI in overall image quality, anatomical detail display, lesion conspicuity, artifact, and distortion reduction, consistent with previous research [16, 18, 19]. Furthermore, we observed that FOCUS DWI significantly improved the shadowing caused by fat signals in conventional DWI, as shown in Fig 4. This improvement could be attributed to the special gradient design and sequence parameters of FOCUS DWI, which mitigate the impact of fat signals on the images, thereby enhancing the overall image quality.

While FOCUS DWI reduced image distortion and blurring, the reduction in the phase-encoding direction might lead to a corresponding decrease in the SNR FOV. Previous studies have indicated that images from DWI with a reduced FOV tend to have lower SNR than those from conventional DWI [18, 26]. In this study, the SNR of FOCUS DWI was slightly, but insignificantly, higher than that of conventional DWI. This may be attributed to the fact that FOCUS DWI doesn’t have SNR penalty caused by parallel imaging. However, the SNR of FOCUS DWI significantly increased after implementing DLR, surpassing both FOCUS and conventional DWI. The study also analyzed changes in signal and noise for the three imaging sequences. The results indicated a significantly lower lesion signal for FOCUS and FOCUS-DLR DWI than for conventional DWI, which could be attributed to the reduced FOV in the phase-encoding direction. The background noise in FOCUS-DLR DWI was significantly lower than in conventional and FOCUS DWI, suggesting that the deep learning algorithm reduced the background noise, thereby improving the overall image SNR.

The ADC values of FOCUS DWI are debated. They can be influenced by factors such as magnetic field strength, choice of b-values, and partial volume effects. Some studies suggested that FOCUS DWI, with its higher spatial resolution and ability to reduce partial volume effects, reflects the lesion condition through its ADC values more accurately [16, 43]. Previous research found no significant difference in ADC values between DWI with a reduced FOV and conventional DWI [16, 18]. However, Feng et al. found that the ADC values of FOCUS DWI were significantly higher than those of conventional DWI [14]. This study found no significant differences in the ADC values among modalities, consistent with previous reports [16, 18]. These results suggest that FOCUS and FOCUS-DLR DWI did not alter the lesion ADC values.

Our study had several limitations. First, the sample size of this study was small and it was a single-center study, potentially introducing selection bias. Future multicenter research with a larger sample is needed for broader generalization. Second, all scans were conducted on a single MRI system. The generalizability of the findings to other MRI platforms remains uncertain and requires further validation. Third, image assessment relied on only two independent readers, lacking diversity in evaluation methods. Additional research with a larger panel of readers is warranted for a more comprehensive evaluation. Lastly, our study did not investigate the influence of breast density on image quality, an important area for future exploration.

## Conclusion

Our findings indicate that FOCUS-DLR DWI produces images superior to conventional DWI without affecting the ADC, enhancing its applicability in clinical practice. DLR is a new direction for breast DWI optimization. FOCUS-DLR DWI offering a promising approach for breast cancer assessment.

## Supporting information

S1 Table Qualitative image evaluation criteria. (DOCX)

S2 Table κ values for inter-observer consistency for qualitative metrics of conventional, FOCUS, and FOCUS-DLR DWI. (DOCX)

## Abbreviations

ADC: apparent diffusion coefficient
CNR: contrast-to-noise ratio
DLR: deep-learning-based reconstruction
DWI: diffusion-weighted imaging
FOCUS: field-of-view optimized and constrained undistorted single-shot
FOV: field-of-view
ICC: intraclass correlation coefficient
ROI: region-of-interest
SD: standard deviation
SD_background_: standard deviation of the background region-of-interest signal intensity
SI: signal intensity
SI_lesion_: signal intensity in the lesion
SNR: signal-to-noise ratio
