# Supplementary material for: Exploring the feasibility of FOCUS DWI with deep learning reconstruction for breast cancer diagnosis: A comparative study with conventional DWI
Source: PLoS One. 2024 Oct 31;19(10):e0313011. doi: 10.1371/journal.pone.0313011 (PMC11527270; doi:10.1371/journal.pone.0313011)
Supplement: S1 Table — (DOCX) [file pone.0313011.s001.docx]

**Supplementary Table 1.** Qualitative image evaluation criteria

| Parameter | Evaluation Criteria and Scoring Standards |
| --- | --- |
| Overall image quality | 1: Very poor; not suitable for diagnosis |
|  | 2: Poor; significantly affects diagnosis |
|  | 3: Fair; has an impact on diagnosis |
|  | 4: Good; suitable for diagnosis |
|  | 5: Excellent; clear structure, suitable for diagnosis |
| Display of anatomical details | 1: Very poor; almost impossible to identify any breast subtle structures or anatomical features and potential lesions |
|  | 2: Poor; most breast structures, subtle features, and potential lesions are unclear and difficult to recognize |
|  | 3: Fair; breast structures, subtle features, and potential lesions are unclear, and might have some blur or unclear areas requiring further observation for identification |
|  | 4: High; anatomical details of breast structures and potential lesions are relatively clear, most subtle structures and features are visible, with a few details possibly slightly blurry |
|  | 5: Excellent; breast tissue structure and lesions are clearly visible, and various subtle structures and anatomical features are easily recognizable |
| Lesion conspicuity | 1: Very poor; lesion difficult to recognize, unsuitable for diagnosis |
|  | 2: Poor; partially visible lesions, significantly affects the diagnosis |
|  | 3: Fair; overall lesion visibility is fair, but edges are blurred |
|  | 4: Good; overall lesion visibility is good and relatively clear |
|  | 5: Excellent; clear lesion visibility, with clearly visible edges |
| Artifacts | 1: Very poor; significant image artifacts, unsuitable for diagnosis |
|  | 2: Poor; relatively noticeable image artifacts, affecting the diagnosis |
|  | 3: Fair; moderate image artifacts, image can be used for diagnosis |
|  | 4: Good; slight image artifacts, not affecting diagnosis |
|  | 5: Excellent; no image artifacts |
| Geometric distortions | 1: Very poor; severe image distortion, unusable for diagnosis |
|  | 2: Poor; the image is mostly distorted, unsuitable for diagnosis |
|  | 3: Fair; partial image distortion |
|  | 4: Good; slight image distortion, does not affect lesion display, suitable for diagnosis |
|  | 5: Excellent; no image distortion |
