# Supplementary material for: Exploring the feasibility of FOCUS DWI with deep learning reconstruction for breast cancer diagnosis: A comparative study with conventional DWI
Source: PLoS One. 2024 Oct 31;19(10):e0313011. doi: 10.1371/journal.pone.0313011 (PMC11527270; doi:10.1371/journal.pone.0313011)
Supplement: S2 Table — (DOCX) [file pone.0313011.s002.docx]

**Supplementary Table 2.** κ values for inter-observer consistency for qualitative metrics of conventional, FOCUS, and FOCUS-DLR DWI

|  | Conventional DWI | FOCUS DWI | FOCUS-DLR DWI |
| --- | --- | --- | --- |
| Overall image quality | 0.232 (*P* < 0.001) | 0.635 (*P* < 0.001) | 0.787 (*P* < 0.001) |
| Display of anatomical details | 0.335 (*P* = 0.016) | 0.638 (*P* < 0.001) | 0.777 (*P* < 0.001) |
| Lesion conspicuity | 0.315 (*P* = 0.007) | 0.657 (*P* < 0.001) | 0.859 (*P* < 0.001) |
| Artifacts | 0.519 (*P* < 0.001) | 0.832 (*P* < 0.001) | 0.855 (*P* < 0.001) |
| Geometric distortions | 0.662 (*P* < 0.001) | 0.701 (*P* < 0.001) | 0.871 (*P* < 0.001) |

DWI, diffusion-weighted imaging; DLR, deep-learning-based reconstruction; FOCUS, field-of-view optimized and constrained undistorted single-shot.
